# Hyaluronan synthases (HAS1-3) and hyaluronidases (HYAL1-2) in the accumulation of hyaluronan in endometrioid endometrial carcinoma

**DOI:** 10.1186/1471-2407-10-512

**Published:** 2010-09-27

**Authors:** Timo K Nykopp, Kirsi Rilla, Markku I Tammi, Raija H Tammi, Reijo Sironen, Kirsi Hämäläinen, Veli-Matti Kosma, Seppo Heinonen, Maarit Anttila

**Affiliations:** 1 Institute of Clinical Medicine, Department of Pathology and Forensic Medicine, University of Eastern Finland and Kuopio University Hospital, Kuopio, Finland; 2 Institute of Biomedicine, Department of Anatomy, University of Eastern Finland, Kuopio, Finland; 3 Institute of Clinical Medicine, Department of Obstetrics and Gynecology, University of Eastern Finland and Kuopio University Hospital, Kuopio Finland

## Abstract

**Background:**

Hyaluronan accumulation correlates with the degree of malignancy in many solid tumor types, including malignant endometrial carcinomas. To elucidate the mechanism of hyaluronan accumulation, we examined the expression levels of the hyaluronan synthases (*HAS1*, *HAS2 *and *HAS3*) and hyaluronidases (*HYAL1 *and *HYAL2*), and correlated them with hyaluronan content and HAS1-3 immunoreactivity.

**Methods:**

A total of 35 endometrial tissue biopsies from 35 patients, including proliferative and secretory endometrium (n = 10), post-menopausal proliferative endometrium (n = 5), complex atypical hyperplasia (n = 4), grade 1 (n = 8) and grade 2 + 3 (n = 8) endometrioid adenocarcinomas were divided for gene expression by real-time RT-PCR, and paraffin embedded blocks for hyaluronan and HAS1-3 cytochemistry.

**Results:**

The mRNA levels of *HAS1-3 *were not consistently changed, while the immunoreactivity of all HAS proteins was increased in the cancer epithelium. Interestingly, *HAS3 *mRNA, but not HAS3 immunoreactivity, was increased in post-menopausal endometrium compared to normal endometrium (p = 0.003). The median of *HYAL1 *mRNA was 10-fold and 15-fold lower in both grade 1 and grade 2+3 endometrioid endometrial cancers, as compared to normal endometrium (p = 0.004-0.006), and post-menopausal endometrium (p = 0.002), respectively. *HYAL2 *mRNA was also reduced in cancer (p = 0.02) and correlated with *HYAL1* (r = 0.8, p = 0.0001). There was an inverse correlation between *HYAL1 *mRNA and the epithelial hyaluronan staining intensity (r = -0.6; P = 0.001).

**Conclusion:**

The results indicated that *HYAL1 *and *HYAL2 *were coexpressed and significantly downregulated in endometrioid endometrial cancer and correlated with the accumulation of hyaluronan. While immunoreactivity for HASs increased in the cancer cells, tumor mRNA levels for *HAS*s were not changed, suggesting that reduced turnover of HAS protein may also have contributed to the accumulation of hyaluronan.

## Background

Cancer of the endometrium is the most common malignant tumor of the female genital tract and it typically affects postmenopausal women [1,2] The prognosis of endometrial cancer is generally good, since the age-adjusted 5-year overall survival is 82% [3]. Although most patients are diagnosed at an early stage, i.e. disease confined to the uterus, still 20% of the cancers recur after primary treatment. Adjuvant treatment does not prolong the overall survival, maybe because of inadequate patient selection. Therefore, new prognostic markers are needed. Molecular markers in endometrial cancer are still rather poorly defined [4].

It has been commonly recognized that development of human neoplasia is accompanied by changes in the extracellular matrix (ECM) which is particularly important in regulating tumor dissemination [5]. The glycosaminoglycan hyaluronic acid/hyaluronan (HA) is a ubiquitous component of the extracellular matrix (ECM).

Hyaluronan is an independent, unfavorable prognostic factor in another gynaecological malignancy, epithelial ovarian cancer [6], and a number of other malignancies [7,8]. Hyaluronan and its receptor CD44 are both involved in the development and progression of endometrial cancer [9].

Hyaluronan can be produced in mammals by three hyaluronan synthase isoenzymes: HAS1, HAS2 and HAS3 [10]. *HAS *mRNA levels often correspond to the rate of hyaluronan synthesis, and are known to influence the content of hyaluronan in transplanted tumors [11]. Therefore, upregulation of *HAS *expression can contribute to the hyaluronan accumulation in tissues, and promote tumor growth and metastasis in experimental animals, in particular when coexpressed with hyaluronidase [12,13].

The catabolism of hyaluronan is more complex process [14]. Hyaluronan in the extracellular matrix can be partially fragmented by hyaluronidase activity or oxygen free radicals, and diffuse away through lymph. Alternatively, hyaluronan can be taken up by adjacent cells and be subject to lysosomal degradation in the tissue of origin [15]. The rate of hyaluronan catabolism may therefore be contributed by the formation of oxygen free radicals, access to lymph, local uptake by cells, and hyaluronidases.

There are 6 hyaluronidases in the human genome, two of them (*HYAL1 *and *HYAL2*) are ubiquitous and characterized at protein level [16]. *HYAL1 *and *HYAL2 *have been shown to inhibit tumor growth *in vivo*, and it has been suggested that these two genes have major roles in the microenvironment of tumor cells [17]. Recent findings have suggested that depending on its concentration, *HYAL1 *can function either as a tumor promoter or as a suppressor [18].

The major transcript of *HYAL3 *is enzymatically inactive and appears to have only a supportive role in *HYAL 1 *expression [19]. *HYAL 3 *knockout mice do not display any evidence of hyaluronan accumulation [20]. Very little is known about HYAL4, but its expression is limited, and it might be a chondroitinase rather than hyaluronidase [16,21]. The expression of the *SPAM1 *gene-encoded PH20 hyaluronidase is almost exclusively detected in testis and sperm, and shows activity in higher pH.

In an invasive bladder cancer cell line, blocking of *HYAL1 *expression decreases tumor growth, inhibits tumor infiltration and decreases microvessel density [22]. Increased hyaluronidase expression has also been reported in prostate [18] and colon cancer[14], and in breast tumor metastases [23]. In contrast, recent findings have shown that the expression of *HYAL1 *and *HYAL2 *genes is significantly decreased in lung and kidney cancer samples [17]. Also experimental overexpression of *HYAL1 *in a rat colon carcinoma cell line inhibits tumor growth and generates necrotic tumors [11].

We have found that the median concentration of hyaluronan is increased in malignant ovarian tumors without hyaluronidase activation [24]. In further studies we have shown that significantly decreased *HYAL1 *expression correlates with decreased hyaluronidase activity and elevated hyaluronan content of the tumors, while *HAS *expression was not as consistently associated to the accumulation of hyaluronan [25].

In this study we found that in the most common gynaecological malignancy, endometrial cancer, the accumulation of hyaluronan is also associated with decreased expression of hyaluronidase genes. Blocking the accumulation of hyaluronan might offer a new way of fighting against these diseases

## Methods

### Patients

A total of 35 endometrial tissue specimens from 35 patients were divided into 5 groups: proliferative and secretory endometrium (n = 10), post-menopausal proliferative endometrium (n = 5), complex atypical hyperplasia (n = 4), grade 1 (n = 8) and grade 2+3 (n = 8) endometrioid adenocarcinomas (Table 1). The normal endometrium tissue specimens were obtained from hysterectomies for nonmalignant diseases (e.g. leiomyoma or prolapse of uterus). The malignant tumors of endometrium were staged according to FIGO. The ethical committee of the Kuopio University Hospital has approved the study protocol and patients signed the informed consent.

**Table 1:** Clinicopathological data of the tissue samples

Tissue type	Histology	No. Patients	**Age at diagnosis**^**†**^	FIGO stage		
				IB	IC	II
Normal endometrium	Proliferating	4	42 (42-45)			
	Secreting	6	48 (41-52)			
Postmenopausal	Proliferating	5	69 (57-75)			
Hyperplastic endometrium	Complex atypical	4	52 (45-68)			
Malignant endometrium						
Endometrioid adenocarcinoma	Grade I	8	66 (46-76)	4	3	1
	Grade II	4	70 (63-81)	2	1	1
	Grade III	4	62 (41-81)	1	2	2
All patients		35				

^† ^median (range)

### Histology

Histological typing and grading were done according to the WHO classification [26,27]. Grade 2 and 3 cancers were combined into one subgroup.

### Tissue samples

The tissue specimens collected in the operation room were prepared and evaluated by an experienced pathologist (KH). All the samples were collected and handled identically. Aliquots of the tissues were 1) placed in RNAlater^® ^(Ambion, Austin, TX) for mRNA analyses; 2) fixed in 10% buffered formalin and embedded in paraffin.

### RNA Extraction and cDNA Preparation

Samples were stored at -80°C until RNA preparation. The samples were frozen by liquid nitrogen and pulverized under pressure using a stainless steel cylinder and a piston. Total RNA was isolated using Trizol^® ^Reagent (Invitrogen) according to manufacturer's protocol, quantified spectrophotometrically and its integrity confirmed by agarose electrophoresis, based on the appearance of the 18S and 28S RNA bands. First strand cDNA was synthesized from 2.5 μg of total RNA using High-Capacity cDNA Archive kit (Applied Biosystems, Foster City, CA) according to manufacturer's protocol in a final volume of 50 μl.

### Quantitative real-time RT-PCR

Real-time gene expression analysis of all target genes (*HYAL1*, *HYAL2*, *HAS1-3*) was performed using TaqMan^® ^Gene Expression Assays (Applied Biosystems) according to manufacturer's instructions and as described previously [25]. The assay numbers for these genes were as follows: Hs00201046_m1 (HYAL1); Hs00186841_m1 (HYAL2); Hs00758053_m1 (HAS1); Hs00193435_m1 (HAS2); Hs00193436_m1 (HAS3); Hs99999909_m1 (HPRT).

The HPRT1 gene we used for normalization was an accurate reference for the quantitative gene expression assays in clinical tumor samples [28]. Relative gene expression values were calculated as the ratio between the target gene and HPRT1, obtained for each sample from the standard curves.

### Staining of HASs

The HAS immunostainings were performed as described previously [25]. Shortly, antigen retrieval was performed for HAS1-3 staining by boiling for 3 × 5 min in a citrate buffer. Thereafter sections were treated for 5 min with 1% H_2_O_2 _to block endogenous peroxidise activity and incubated in 1% bovine serum albumin (BSA) in PBS for 30 min to block nonspecific binding. The sections were incubated overnight at 4°C with polyclonal antibodies for HAS1 (2 μg/ml, sc-34021, Santa Cruz Biotechnology, inc., Santa Cruz, CA), HAS2 (2 μg/ml, sc-34067, Santa Cruz) or HAS3 (2 μg/ml sc-34204, Santa Cruz), diluted in 1% BSA. Sections were incubated for 1 hour with biotinylated antigoat antibody (1:1000, Vector Laboratories). The bound antibodies were visualized with the avidin-biotin peroxidase method (1:200, Vectastain Kit, Vector Laboratories), yielding a brown reaction product. The sections were counterstained with Mayer's hematoxylin. The staining intensity of HAS1, HAS2 and HAS3 was graded into three categories in the epithelium: negative (n.d.), weak and moderate, and into two categories in the stroma: negative (n.d.) or weak. The percentage of positive area for each HAS was estimated both in stroma and epithelium.

### Staining of Hyaluronan

Deparaffinized 5-μm tissue sections were stained for hyaluronan with our own preparation of biotinylated hyaluronan-binding complex (bHABC) as described in detail previously [24]. All samples were scored by an observer unaware of the clinical data (M.A.). The intensity of hyaluronan positivity in epithelium and in stroma was graded into three categories: 1 (weak); 2 (moderate); and 3 (strong) and the area percentage of the strongest hyaluronan expression in the whole tumor section was evaluated separately in epithelium and stroma.

### Statistical methods

Statistical analyses were carried out using SPSS 16.0 for Windows (SPSS, Chicago, IL). Differences between the patient groups were first analysed by using a non-parametric Kruskal-Wallis test, and when found significant a non-parametric Mann - Whitney U-test was used for further comparisons between the patient groups. Correlations between gene expression data and hyaluronan staining and immunostaining scores were analysed by using the Spearman's correlation test. A Chi-square test was used to analyse the association of hyaluronan staining and immunostaining scores. We considered p-value ≤ 0.01 as statistically significant.

## Results

### Expression of *HAS1*, *HAS2 *and *HAS3*

**Figure 1:**
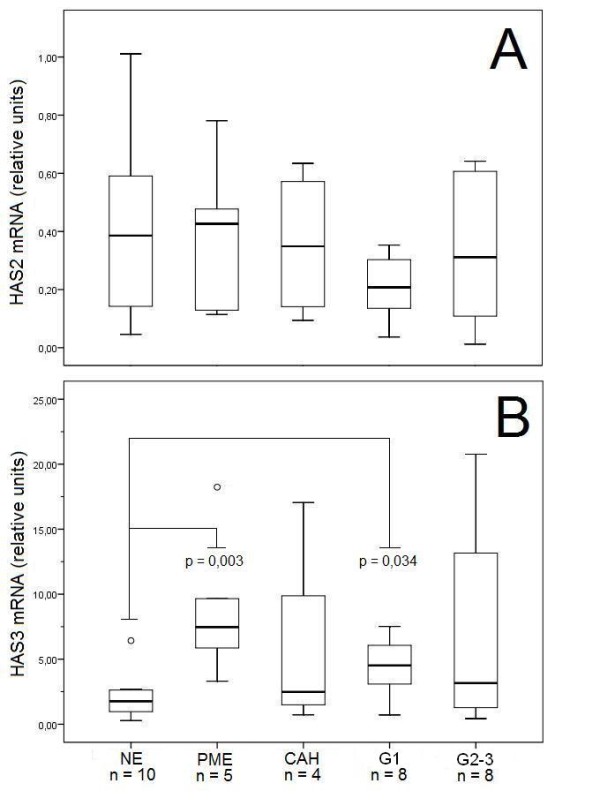
***HAS 2-3 *mRNA expression in human endometrium and its lesions**. Relative mRNA levels of A) *HAS2 *and B) *HAS3 *in different tissues. The boxes show the ranges between 25th and 75th percentiles, with a horizontal line at the median value. The whiskers extend to the 10th and 90th percentiles. The open circles represent the outlier values. NE = normal pre-menopausal endometrium, PME = post-menopausal proliferative endometrium, CAH = complex atypical hyperplasia, G1 = grade 1 endometrioid endometrial adenocarcinoma, G2 + 3 = grade 2 + 3 endometrioid endometrial adenocarcinoma.

mRNA from normal endometrium and different tissue lesions were analyzed by real-time RT-PCR for the hyaluronan synthases *HAS1*, *HAS2*, and *HAS3 *(figure 1). Transcripts of *HAS1 *were detected at such a low level that reliable quantitation was not consistently possible. Expression of *HAS2 *was not significantly changed in malignant tumors or the postmenopausal or hyperplastic tissues as compared to normal endometrium (figure 1A). On the other hand *HAS3 *expression was increased over 4-fold in post-menopausal endometrium (p = 0.003) compared to pre-menopausal endometrium. We also noticed that *HAS3 *expression was also elevated (1.5-fold increase) in grade 1 malignant tumors compared to normal endometrium, this finding being borderline significant (p = 0.033) (figure 1B).

### Expression of *HYAL1 *and *HYAL2*

**Figure 2:**
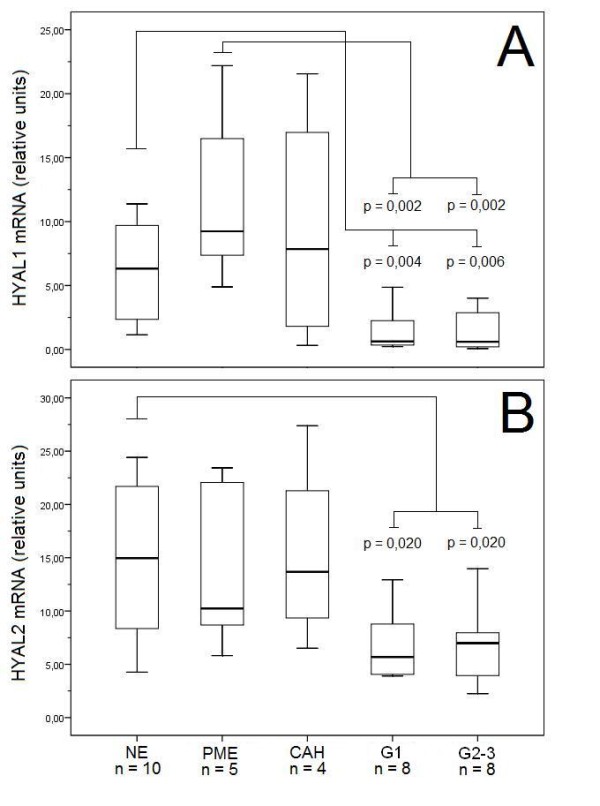
***HYAL 1-2 *mRNA expression in human endometrium and its lesions**. Relative mRNA levels of A) *HYAL1 *and B) *HYAL2 *in different tissues. The boxes show the ranges between 25th and 75th percentiles, with a horizontal line at the median value. The whiskers extend to the 10th and 90th percentiles. NE = normal pre-menopausal endometrium, PME = post-menopausal proliferative endometrium, CAH = complex atypical hyperplasia, G1 = grade 1 endometrioid endometrial adenocarcinoma, G2 + 3 = grade 2 + 3 endometrioid endometrial adenocarcinoma.

Since the two ubiquitous hyaluronidases, *HYAL1 *and *HYAL2*, were likely to account for most or all of the hyaluronidase activity, we quantified their mRNA levels by real-time RT-PCR (figure 2). There was a significant decline in *HYAL1 *expression from normal endometrium to the cancers (Kruskal Wallis p = 0.002). A 10-fold higher expression of median *HYAL1 *mRNA expression was found in normal endometrium as compared to both grade 1 and grade 2 + 3 malignant tumors, and over 15-fold higher values were seen in normal post-menopausal endometrium (figure 2A). Similar trend was noticed between *HYAL2 *expression in grade 1 and grade 2 + 3 cancers compared to normal endometrium, this difference being borderline significant (figure 2B). There was also a strong and statistically significant correlation between *HYAL1 *and *HYAL2 *expressions (r = 0.8; p < 0.0001)

### HAS immunostainings

**Figure 3:**
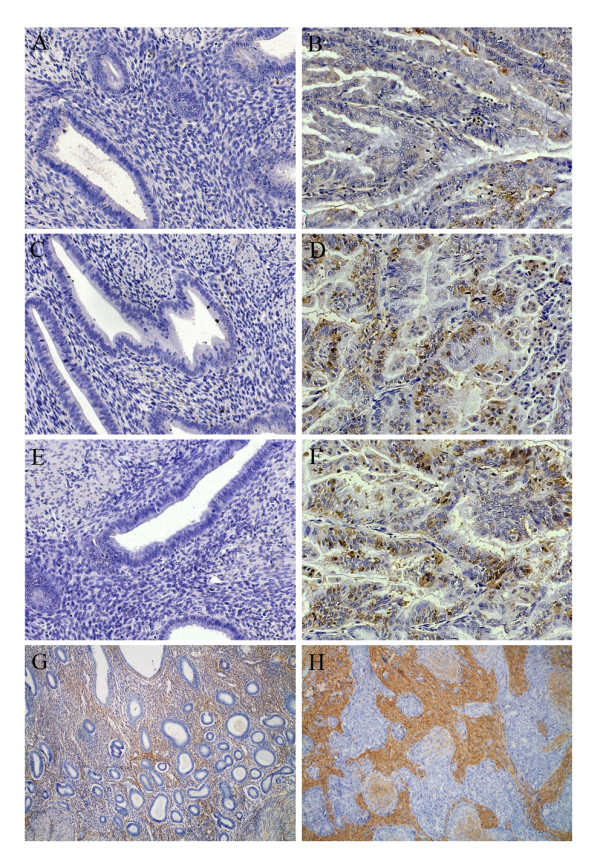
**HAS 1-3 immunoreactivity and HA-staining in normal and neoplastic human endometrium**. Immunostaining of HAS1 (A, B), HAS2 (C, D), HAS3 (E, F) and HA (G, H) in normal human endometrium (A, C, E, G) and in grade 2 endometrioid endometrial carcinoma tissue sections (B, D, F, H). The brown color (DAB) indicates HAS (A-F) or HA (G, H), and blue color (haematoxylin) indicates nuclei. A-F: 200× original magnification. G, H: 100× original magnification.

The epithelial intensities of HAS1 (figure 3A,B), HAS2 (figure 3C,D) and HAS3 (figure 3E,F) immunostainings were significantly stronger in endometrial tumors compared to normal endometrium (p = 0.001, p = 0.004 and p = 0.003, respectively) (table 2). However, the staining intensities did not correlate with tumor grade. Epithelial hyaluronan staining intensity was associated with HAS2 epithelial staining intensity (p = 0.009). No significant correlations were found between the mRNA levels and immunohistochemical protein scores of HAS1-3. On the other hand, an inverse correlation of *HYAL1 *expression with HAS3 epithelial staining intensity was found (r = -0.5, p = 0.004).

**Table 2:** Immunostaining of HAS1, HAS2 and HAS3 in human endometrium and its lesions

		HAS1 staining intensity*					HAS2 staining intensity*					HAS3 staining intensity*				
		Epithelium			Stroma		Epithelium			Stroma		Epithelium			Stroma	
		n.d.	weak	moderate	n.d.	weak	n.d.	weak	moderate	n.d.	weak	n.d.	weak	moderate	n.d.	weak
Normal n = 10		4	2 (10-25%)	4 (10-50%)	7	3 (5-50%)	7	3 (5-10%)	0	8	2 (25%)	5	5 (10-20%)	0	9	1(10%)
Proliferating pm^† ^n = 5		5	0	0	5	0	5	0	0	1	4 (5%)	5	0	0	5	0
Hyperplasia ^# ^n = 4		1	3 (5-25%)	0	3	1 (10%)	1	2 (10-25%)	1 (50%)	3	1 (10%)	2	2 (10%)	0	3	1(25%)
Grade 1 ^‡ ^n = 8		0	5 (5-50%)	3 (25-50%)	6	2 (5%)	2	5 (5-25)	1 (50%)	8	0	3	3 (5-25%)	2 (25-50%)	8	0
Grade 2 + 3 ^‡ ^n = 8		0	2 (25-50%)	6 (5-50%)	8	0	0	5 (5-50%)	3 (5-50)	7	1 (5%)	0	3 (10-20%)	5 (10-25%)	8	0

* num. of samples with % of positive cells (% in median; range when necessary)

n.d. = not detected/negative

^# ^complex atypical hyperplasia

^†^pm = post-menopausal

‡ endometrioid endometrial adenocarcinoma

### Hyaluronan content

The level of hyaluronan accumulation in the present set of lesions was scored from tissue sections using a biotinylated probe that specifically binds hyaluronan [6] (figure 3G,H). This histological assay closely correlates with biochemical quantitation of hyaluronan [24]. Epithelial and also stromal hyaluronan intensity score was significantly elevated (p = 0.0001 and p = 0.006 respectively) in the endometrioid endometrial tumors compared to normal endometrium (table 3).

**Table 3:** Hyaluronan staining in human endometrium and its lesions

	HA-staining intensity				HA-staining intensity			
	Epithelium*				Stroma*			
	n.d.	weak	moderate	strong	n.d.	weak	moderate	strong
Normal n = 9	9	0	0	0	0	0	9 (60%; 10-90%)	0
Proliferating pm^† ^n = 5	5	0	0	0	0	3	1 (30%)	1 (100%)
Hyperplasia ^# ^n = 2	0	1 (5%)	1 (40%)	0	0	1 (40%)	0	1 (30%)
Grade 1 ^‡ ^n = 8	1	0	5 (2-5%)	2 (5-10%)	0	0	5 (70%; 20-80%)	3 (80%; 50-100%)
Grade 2+3 ^‡ ^n = 8	1	0	4 (15%; 10-60%)	3 (30%; 20-30%)	0	0	2 (50-80%)	6 (80%; 20-90%)

* num. of samples with % of positive cells (% in median; range when necessary)

n.d. = not detected/negative

^# ^complex atypical hyperplasia

^†^pm = post-menopausal

‡ endometrioid endometrial adenocarcinoma

### Relationship between hyaluronan accumulation and *HAS *and *HYAL *mRNA levels

No significant correlations were found between the level of *HAS2 *or *HAS3 *mRNA and the hyaluronan content. Instead, *HYAL1 *transcript levels showed a significant inverse correlation with epithelial (r = -0.6, p = 0.001) and stromal hyaluronan staining score (r = -0.4 p = 0.01). *HYAL2 *transcript levels also correlated with epithelial hyaluronan staining score (r = -0.4 p = 0.01).

## Discussion

Hyaluronan synthase and hyaluronidase mRNA levels were quantitated for the first time in a set of sample groups from normal endometrium, post-menopausal endometrium, endometrial hyperplasia and endometrioid endometrial tumors.

### *HAS *mRNA expression and immunohistochemistry in endometrioid endometrial cancer

Except for a trend of *HAS3 *increase in grade 1 carcinomas, we did not find a clear pattern of increased *HAS1-3 *mRNA in endometrial cancer, as compared to normal endometrium. This finding is somewhat similar to the enhanced *HAS3 *mRNA level observed in benign, but not in malignant ovarian tumors [25]. On the other hand, *HAS3 *mRNA was increased in proliferating post-menopausal endometrium, and has been suggested to promote the growth of bladder carcinoma cells [29]. Nevertheless, our results suggested that transcriptional upregulation of *HAS *gene expression was not the main contributor to the increased hyaluronan content of these tumors.

Despite minor changes in mRNAs, the immunoreactivity for all HASs was stronger in cancer cells compared to normal endometrium, although the density of the stainings did not significantly correlate with tumor grade. A similar discordance between the levels of *HAS *mRNA and HAS immunoreactivity was earlier found in ovarian cancer [25]. Curiously, the statistical significance for the cancer-associated increase of HAS immunoreactivity was strongest for HAS1 although real-time RT-PCR suggested very low transcription of this gene. As in the present study, Yabushita et al. [30] found that HAS1 immunoreactivity showed a strong association with endometrial cancer.

*HAS2 *is the only *HAS *gene which deletion causes a clear (lethal) phenotype [31], and it has been suggested to be most important for hyaluronan synthesis. In line with this idea, the immunohistochemical signal of epithelial HAS2 correlated with the staining score of epithelial HA.

Since the *HAS *gene expressions in tumor tissues poorly correlated with the content of the respective HAS proteins, as suggested by immunocytochemistry, the turnover of the HAS proteins should become slower in cancer cells. The time of functional work life of the HAS proteins in tissues was not known, but if extended, it may have had a major influence on hyaluronan synthesis.

### Decreased expression of *HYAL1 *and *HYAL2 *mRNA and association to hyaluronan accumulation

The present findings of decreased *HYAL1 *and *HYAL2 *mRNA in endometrial cancer are consistent with the results we have recently shown in ovarian cancer [25]. Our findings are also similar to those of lung and kidney cancer samples in which a major down-regulation of *HYAL1 *and *HYAL2 *genes has been shown [17]. Our findings in endometrial and ovarian cancer are in contrast to reports on prostate and bladder tumors in which increased HYAL1 protein and mRNA expression is associated with advanced disease and unfavorable prognosis [32,18,22]. One hypothesis to solve this contradiction between different types of cancers is that malignancies arising from different cell types use different strategies to progress and evolve. The relative importance of the opposite roles of hyaluronidase function in a particular type of cancer probably determines the outcome. The expression level is also important since *HYAL1 *can either promote or suppress malignant growth in a single cell type, depending on the resulting enzyme activity [18].

### *HYAL1*, a tumor suppressor

*HYAL1 *and *HYAL2 *genes are located in 3p21.3 chromosome region, where allelic imbalance is frequent [33,14]. In the present study we noticed a strong correlation between *HYAL1 *and *HYAL2 *mRNA expression. It is possible that in endometrial cancer the *HYAL *mRNA expression depends on the status of the chromosome 3p21.3 locus, and the decreased expression of *HYAL1 *and *HYAL2 *may be explained by concomitant deletions of these closely mapped genes. One theory to explain why decreased expression of hyaluronidase genes could lead to the development of cancer is *HYAL1 *and its role in apoptosis. High HYAL1 activity can result in apoptosis by increasing the expression of WOX1 (WW domain-containing oxidoreductase, WWOX) [22]. WOX1 causes mitochondrial permeabilization and is an essential partner of p53 in cell death [34]. Hyaluronidase can also cause apoptosis by inducing NAD+-linked 15-hydroxyprostaglandin dehydrogenase (15-PDGH), an enzyme that degrades prostaglandins and promotes apoptosis in lung carcinoma cells [35]. Furthermore, the high molecular mass hyaluronan that occupies cell surface CD44 receptors maintains p-Akt and PI3K dependent signals that prevent cancer cell apoptosis, while hyaluronidase, and the oligosaccharides created by hyaluronidase, block these cell survival signals [36]. It has also been recently demonstrated that ectopic expression of all three different somatic hyaluronidases (*HYAL1, HYAL2 *and *HYAL3*) induce granulosa cell apoptosis [37].

## Conclusion

The results indicated that *HYAL1 *and *HYAL2 *were coexpressed and significantly downregulated in endometrioid endometrial cancer and correlated with the accumulation of hyaluronan. The expression of *HAS1-3 mRNA *is not elevated in endometrioid endometrial cancer while their immunoreactivity is elevated, suggesting cancer-associated changes in HAS protein turnover.

## Abbrevations

HYAL: hyaluronidase; HAS: hyaluronan synthase; FIGO: International Federation of Gynecologists and Obstetrics.

## Competing interests

The authors declare that they have no competing interests.

## Authors' contributions

TN performed the RNA extraction and RT-QPCR analyses, performed statistical analyses and drafted the manuscript. KR analysed the HAS staining and contributed to the manuscript. MT participated in design of the study and helped to draft the manuscript. KH contributed to pathological analysis of the tissue samples and helped to draft the manuscript. RT, RS, V-MK, and SH participated in design of the study and helped to draft the manuscript. MA conceived of the study, and participated in its design and coordination and helped to draft the manuscript.

All authors read and approved the final manuscript.

## Pre-publication history

The pre-publication history for this paper can be accessed here:

http://www.biomedcentral.com/1471-2407/10/512/prepub
